# Recent Trends in Hospitalizations for Infection in the First Year After Heart Transplantation

**DOI:** 10.1016/j.atssr.2025.05.007

**Published:** 2025-06-04

**Authors:** Shi Nan Feng, Alexandra Rizaldi, Atharv Oak, Alice L. Zhou, Armaan F. Akbar, Jessica M. Ruck, Ahmet Kilic

**Affiliations:** 1 Division of Cardiac Surgery, The Johns Hopkins Hospital, Baltimore, Maryland

## Abstract

**Background:**

Infection is a major cause of morbidity and mortality after heart transplantation (HT). Recent trends in post-HT infection demand further investigation, particularly given effects of the coronavirus disease 2019 (COVID-19) pandemic.

**Methods:**

We identified all adult HT recipients in the United States from October 18, 2018, to June 30, 2023, using Organ Procurement and Transplantation Network data. We categorized transplants into 3 eras, accounting for COVID-19: October 2018 to March 2020, March 2020 to March 2022, and after March 2022. Survival was compared using Kaplan-Meier survival analysis and Cox proportional hazards regression. Readmission hospitalizations for infection in the first year after HT were compared using multivariable logistic regression, adjusted for era and donor and recipient characteristics.

**Results:**

Of 13,663 patients who received HT (median age, 57 years; 72.9% men), hospitalization for infection in the first year after transplant was 2.9% (n = 3645) for patients who received a transplant in October 2018 to March 2020, 5.4% (n = 5658) for patients who received a transplant in March 2020 to March 2022 and 11.6% (n = 4360) for patients who received a transplant after March 2022 (*P* < .001). Compared with patients who received a transplant between October 2018 and March 2020, patients who received a transplant during March 2020 to March 2022 (adjusted odds ratio, 1.91; 95% CI, 1.51-2.41) and after March 2022 (adjusted odds ratio, 4.37; 95% CI, 3.30-5.78) eras were more likely to be hospitalized for infection in their first year after HT. After adjusting for covariates, we found no significant difference in the risk of death for recipients who received a transplant from March 2020 to March 2022 (adjusted hazard ratio, 0.86; 95% CI, 0.67-1.11; *P* = .257) or after March 2022 (adjusted hazard ratio, 1.01; 95% CI, 0.73-1.39, *P* = .955) compared with March 2018 to March 2020.

**Conclusions:**

Odds of hospitalization for infection in the first year after HT performed between March 2020 and March 2022 and after March 2022 were 1.91 and 4.37 times as high, respectively, as HT performed between October 2018 and March 2020.

**In Short**

▪ The likelihood of hospitalization for infection within the first year after heart transplantation increased significantly from March 2020 to June 2023 compared with October 2018 to March 2020.
▪ Acute rejection decreased across eras, whereas posttransplant survival did not significantly differ.

For selected patients with advanced heart failure, orthotopic heart transplantation (HT) remains the gold standard therapy to improve quality of life and extend survival.^1^ Infection after HT continues to be a major cause of morbidity and mortality, particularly later in the posttransplant period.^2^ Risk factors for severe outcomes from infection can include individual- and population-level factors, and studies have also shown that certain infections, such as severe acute respiratory syndrome coronavirus 2, can increase the risk of a secondary bacterial coinfection.^3^^,^^4^ Additionally, infection risk can be further amplified in patients who are placed on temporary mechanical circulatory support devices.^5^ Given these complexities, understanding infection burden in HT recipients, particularly in the context of the coronavirus disease 2019 (COVID-19) pandemic, is of timely importance.

Current approaches to induction therapy aim to minimize the risk of allograft rejection from under-immunosuppression while concurrently preventing infection and drug toxicity from over-immunosuppression.^6^ To this degree, achieving a delicate balance between immunosuppression and infection risk is an important consideration with respect to maximizing post-HT survival. Prior studies have characterized the prevalence and outcomes associated with specific types of infections in HT recipients at the institutional level, but analysis is lacking on recent trends in post-HT infection risk on a national scale.

Using national registry data, we aimed to fill this gap by characterizing trends in hospitalizations for infection in the first year after HT across recent time eras, in the context of the COVID-19 pandemic. We also evaluated changes in recipient characteristics, posttransplant outcomes, and survival during these eras.

## Material and Methods

### Data Source

The data provided in this report were sourced from the United Network for Organ Sharing (UNOS), acting as the contractor for the Organ Procurement and Transplantation Network (OPTN). The interpretation and presentation of these data are the authors' responsibility and should not be considered an official stance or interpretation by OPTN or the United States Government. The Johns Hopkins Institutional Review Board determined this study to be exempt from review (IRB00352819, approved December 19, 2022).

### Study Population

Using Scientific Registry of Transplant Recipients data, we first identified all adult (≥18 years) HT recipients without previous or multiorgan transplants between October 18, 2018, and June 30, 2023. October 18, 2018, was selected as the start date for our study period to account for the October 2018 allocation policy change. We categorized transplants into 3 eras to account for both the October 2018 allocation policy change and before, during, and after COVID-19 pandemic contexts. Era 1 included transplants performed between October 18, 2018, and March 2020, era 2 included transplants performed between March 2020 and March 2022, and era 3 included transplants performed between March 2022 and June 30, 2023.

### Statistical Analysis

Normality of variables was assessed using Shapiro-Wilk testing and histogram visualization. We compared donor, recipient, and transplant characteristics by era using the χ^2^ test for categorical variables and the Kruskal-Wallis test for continuous variables. To compare readmission hospitalizations for infection in the first year after HT by era, we performed multivariable logistic regression adjusted for donor and recipient characteristics. Covariates included in the final multivariable models were selected according to clinical relevance and by significance at a level of *P* < .1 on univariate analysis and included era, recipient age, sex, race/ethnicity, body mass index, diabetes, extracorporal membrane oxygenation, ventilator, blood group, days on the waiting list, and induction therapy (antithymocyte globulin and basiliximab); and donor age, sex, and race/ethnicity. The same covariates were used for adjustment in all multivariable models.

For posttransplant outcomes available in Scientific Registry of Transplant Recipients data as binary variables, including 1-year acute rejection, posttransplant dialysis, and posttransplant stroke, we compared proportions by era using χ^2^ or Fisher exact testing for univariate analysis and multivariable logistic regression for multivariate analysis. Posttransplant hospital length of stay was analyzed as a continuous variable and compared between eras using Wilcoxon rank sum testing. Given the highly skewed nature of the data, we did not perform multivariable linear regression on length of stay. All adjusted results are indicated with an “a” before the point estimate type (ie, aHR for adjusted hazard ratio and aOR for adjusted odds ratio). All analyses were performed using Stata 17.0/MP for Windows (StataCorp LLC).

## Results

### Study Population

Of 13,663 patients who received HT (median age, 57 years; men, 72.9%), 3645 patients received a transplant during era 1 (October 18, 2018-March 2020), 5658 patients received a transplant during era 2 (March 2020-March 2022), and 4360 patients received a transplant during era 3 (March 2022-June 2023) (Table 1).

**Table 1:** Characteristics of Heart Transplant Recipients by Era

Recipient Characteristics	Before 03/2020 (n = 3645)	3/2020-03/2022 (n = 5658)	After 3/2022 (n = 4360)	*P* Value
Age, y	56 (46-63)	57 (46-64)	56 (46-62)	<.001
Male sex	2627 (72.1)	4163 (73.6)	3167 (72.6)	<.001
Race/ethnicity				<.001
White	2312 (63.4)	3474 (61.4)	2506 (57.5)	
Black	833 (22.9)	1342 (23.7)	1109 (25.4)	
Hispanic	338 (9.3)	561 (9.9)	509 (11.7)	
Other	162 (4.4)	281 (5.0)	236 (5.4)	
Body mass index, kg/m^2^	28 (24-31)	28 (24-31)	27 (24-31)	<.001
Diabetes	957 (26.3)	1512 (26.7)	1235 (28.3)	<.001
Extracorporal membrane oxygenation	175 (4.8)	323 (5.7)	287 (6.6)	<.001
Ventilator support	84 (2.3)	113 (2.0)	88 (2.0)	.11
Blood type				<.001
A	1468 (40.3)	2195 (38.8)	1660 (38.1)	
AB	190 (5.2)	275 (4.9)	207 (4.7)	
B	566 (15.5)	870 (15.4)	651 (14.9)	
O	1421 (39.0)	2318 (41.0)	1842 (42.2)	
Days on waiting list	42 (10-198)	30 (9-153)	25 (9-110)	<.001
Induction therapy				
Antithymocyte globulin	70 (1.9)	92 (1.6)	54 (1.2)	<.001
Basiliximab	922 (25.3)	1415 (25.0)	1135 (26.0)	<.001
Outcomes				
Acute rejection	743 (20.4)	1029 (18.2)	653 (15.0)	<.001
Dialysis	524 (14.4)	806 (14.2)	696 (16.0)	<.001
Stroke	140 (3.8)	202 (3.6)	197 (4.5)	<.001
Hospital length of stay, d	17 (12-25)	17 (12-25)	17 (12-26)	<.001
Hospitalization for infection (1 year)	99 (2.9)	273 (5.4)	114 (11.6)	<.001

Data are presented as median (interquartile range) or n (%).

Pretransplant extracorporeal membrane oxygenation increased over the study period, from 4.8% (n = 175) of patients in era 1, to 5.7% (n = 323) in era 2, and 6.6% (n = 287) in era 3 (*P* < .001). Meanwhile, median days on the waiting list decreased from 42 days (interquartile range [IQR], 10-198 days) in era 1, to 30 days (IQR, 9-153 days) in era 2, and 25 days (IQR, 9-110 days) in era 3 (*P* < .001). Antithymocyte use decreased from eras 1 to 3 (*P* < .001), whereas basiliximab use decreased between eras 1 and 2 before increasing in era 3 (*P* < .001) (Table 1).

### Posttransplant Outcomes by Era

Frequency of acute rejection decreased across eras, from 20.4% (n = 743) in era 1 to 18.2% (n = 1029) in era 2 and 15.0% (n = 653) in era 3 (*P* < .001) (Table 1). In contrast, posttransplant dialysis increased over the study period after an initial decrease, and posttransplant stroke similarly decreased then increased. Median hospital length of stay was 17 days across eras (Table 1).

Multivariable regression indicated that, compared with era 1, odds of acute rejection were significantly lower in era 2 (aOR, 0.88; 95% CI, 0.79-0.98, *P* = .017) and era 3 (aOR, 0.69; 95% CI, 0.61-0.78; *P* < .001) (Table 2). However, odds of posttransplant dialysis and stroke were not significantly different (Table 2).

**Table 2:** Multivariable Analyses for Postoperative Heart Transplantation Outcomes by Era

Outcome (ref: Before 03/2020)	Odds Ratio^a^	95% CI	*P* Value
Acute rejection			
03/2020-03/2022	0.88	0.79-0.98	.017
After 03/2022	0.69	0.61-0.78	<.001
Posttransplant dialysis			
03/2020-03/2022	0.98	0.87-1.10	.718
After 03/2022	1.13	1.00-1.28	.054
Posttransplant stroke			
03/2020-03/2022	0.91	0.73-1.14	.421
After 03/2022	1.19	0.95-1.49	.126
Hospitalization for infection (ref: Before 03/2020)			
03/2020-03/2022	1.91	1.512.41	<.001
After 03/2022	4.37	3.30-5.78	<.001

a Adjusted for era, recipient age, sex, race/ethnicity, recipient body mass index, diabetes, extracorporeal membrane oxygenation, ventilator, blood group, days on waiting list, and induction therapy (antithymocyte globulin/basiliximab); and donor age, sex, and race/ethnicity.

**Figure 1:**
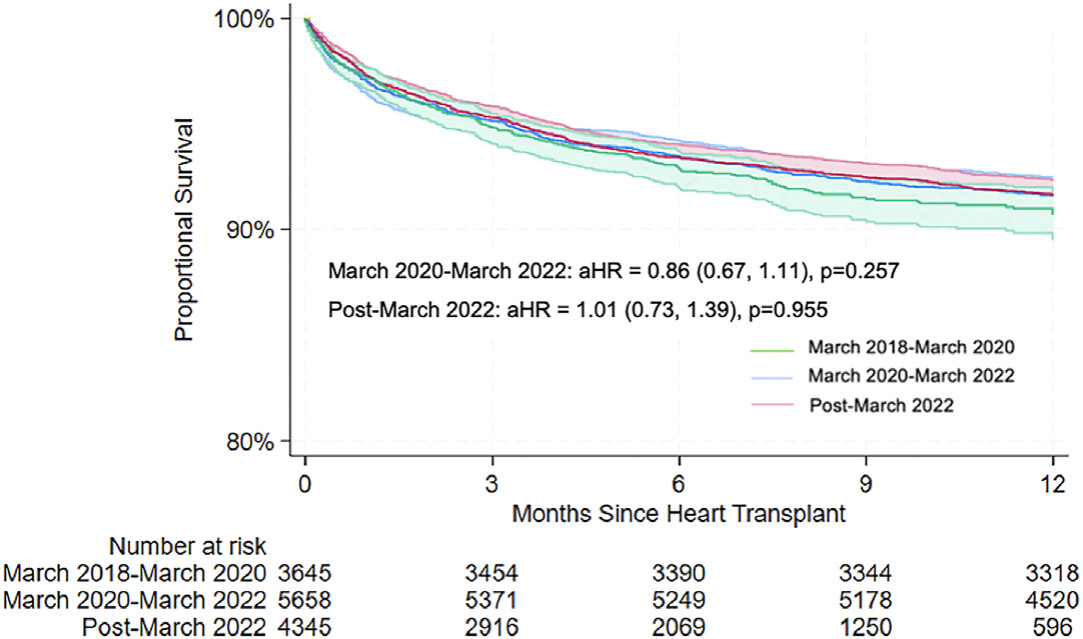
Heart transplant survival at 1 year by era. Shaded area indicates the 95% CI. (aHR, adjusted hazard ratio.)

After adjusting for donor and recipient characteristics, survival analysis showed no significant difference in the risk of death for HT recipients who received a transplant during era 2 (aHR, 0.86; 95% CI, 0.67-1.11; *P* = .257) or era 3 (aHR, 1.01; 95% CI, 0.73-1.39; *P* = .955) compared with era 1 (Figure 1).

### Hospitalization for Infection by Era

**Figure 2:**
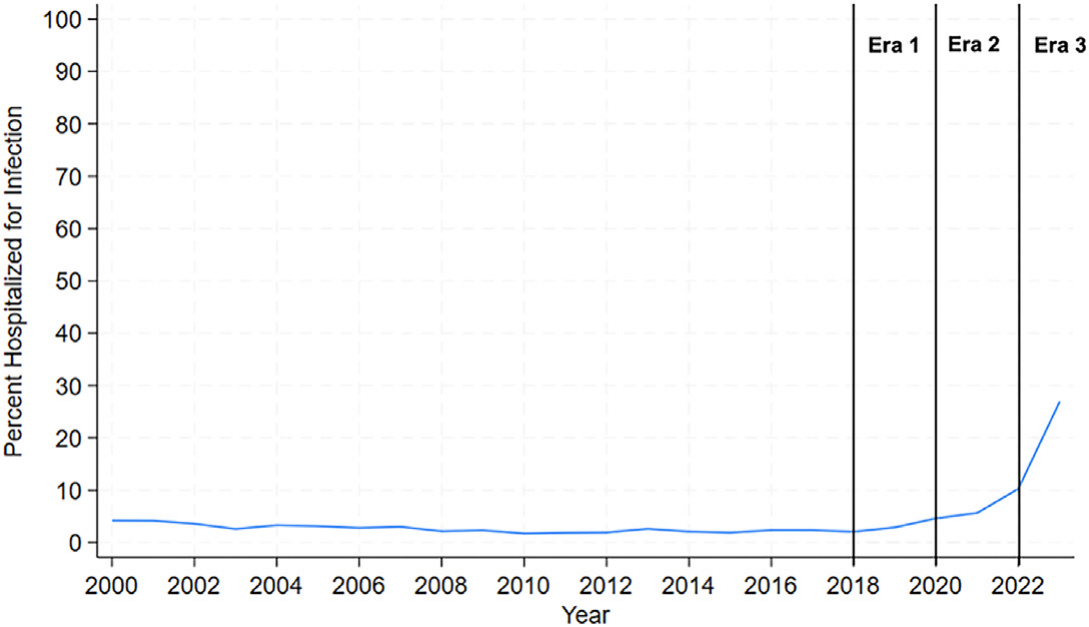
Percentage of heart transplant recipients hospitalized for infection in the first year.

Of the 486 patients hospitalized for infection in their first year after HT during the study period, the hospitalization rate was 2.9% (n = 99) for patients who received a transplant in era 1, 5.4% (n = 273) for patients who received a transplant in era 2, and 11.6% (n = 114) for patients who received a transplant in era 3 (*P* < .001) (Table 1; Figure 2).

Multivariable regression analyses showed that compared with patients who received a transplant in era 1, odds of hospitalization for infection in the first year after HT was 1.91 times as high for patients who received a transplant during era 2 (aOR, 1.91; 95% CI, 1.51-2.41) and 4.37 times as high for transplants performed during era 3 (aOR, 4.37; 95% CI, 3.30-5.78) (*P* < .001) (Table 2).

## Comment

In this national retrospective analysis of HT outcomes from 2018 to 2023, we found that odds of hospitalization for infection in the first year after HT performed between March 2020 and March 2022 and after March 2022 were 1.91 and 4.37 times as high, respectively, as HT performed between October 2018 and March 2020, despite no significant differences in survival during the same time periods.

The significant rise in infection-related hospitalizations likely reflects changes in patient management in recent years, particularly during and after the COVID-19 pandemic. For example, use of temporary mechanical circulatory support devices, which has been associated with increased risk of postoperative infections,^5^^,^^7^, ^8^, ^9^ has risen considerably in recent years. Moreover, patients receiving temporary mechanical circulatory support often require prolonged hospitalization, contributing to infection risk. Changes in the health care landscape due to COVID-19 (ie, residual effects on immune function, staff shortages and backlogs in care, and shifts in immunosuppression use, including calcineurin inhibitors) may have also heightened vulnerability of HT recipients to infections. Improvements in infection detection could have also contributed to increased infection rates. Finally, evolving donor/recipient characteristics, including increased hepatitis C virus–positive donors, longer ischemic times, and post–COVID-19 donor considerations, add complexity to posttransplant immunologic surveillance and likely contributed to infection risk.

Despite the rising incidence of infections, survival rates did not differ significantly across eras. This may suggest that effective strategies for identifying and managing infectious complications could have lessened the effect of infections on overall mortality. Separately, the decrease in the odds of acute rejection during the study period likely reflects factors including advancements in immunosuppression and improved diagnostic tools for early detection. However, the concomitant rise in posttransplant infections highlights a growing concern about the immunologic trade-offs associated with more potent immunosuppression. Developing strategies to optimize immunosuppression while minimizing infection risk is key to improving survival and quality of life in HT recipients.

Our study has several limitations. As a retrospective analysis, we were limited in our ability to derive causal relationships between observed infection trends and recipient factors. Additionally, the OPTN does not include granular data regarding maintenance immunosuppression. We were also unable to measure time between transplant and hospitalization for infection or distinguish the severity or type of infection, including COVID-19. We were limited in our ability to account for specific health care system disruptions or the role of COVID-19 vaccination and unable to evaluate long-term outcomes and survival.

In conclusion, our study revealed a dramatic increase in the risk of post-HT infections during and after the COVID-19 pandemic, with the largest increase seen after March 2022. Although survival outcomes have remained unchanged, the increasing burden of infection-related morbidity underscores the need for further research into infection prevention and management for HT recipients.
